# Louis Pasteur

**Published:** 1895-10-05

**Authors:** 


### Louis Pasteur.

It is not our place to give an account of the life and work of M. Pasteur; pages would be required where we could give but lines, and we would not wish that the homage we render to the memory of so great a man should be measured by the space we can devote to such a topic.

But while we leave others to fulfil the duties of biographers, we feel called upon to draw attention to the wonderful influence which this one man's genius has had not only upon the progress of science, but upon the future of humanity. Facts had long been accumulating, the presence of micro-organisms in certain diseases and in certain forms of fermentation was known, but it was the master mind that saw their *rôle* and was able to point out their world-wide importance.

Far too much has it been the custom of late years to associate the name of Pasteur with his discoveries in regard to hydrophobia. Great as has been the influence of that discovery, it was not there that the real pioneer was shown, but rather in his work in regard to fermentations, to silkworm disease, and to anthrax, work by which the true position of micro-organisms in nature's laboratory was made so manifest. It is with difficulty that one can so throw the mind back for forty years as to realise the conceptions which then were prevalent in regard to many biological questions, nor would it be fair to others to pretend that to one man alone is due the present condition of our knowledge; but it is certain that while many were working parallel with him in the wide fields of plodding investigation opened up by the study of micro-organisms, Pasteur was the pathfinder, the one to give the clue where-ever the trail was lost, to show the way when the scent had failed. Pasteur was no narrow specialist. He was not primarily a man of microbes. It was in the realms of physics and of chemistry that he had made for himself a name and attained a position before he was heard of in relation to the processes of disease; but he turned to biology, as to any other tool, to elucidate a point which he was investigating, and it was thus by bringing biology to bear on what till then had been a chemical problem that he was led to the great generalisation which will always be associated with his name—that all forms of fermentation, including phases of decomposition and putrefaction, the relation of which to fermentation was then unthought of, are due to the action of living organisms. Others had been working in the same field, but it was Pasteur who put his finger on the real secret, linked together all those changes of which fermentation is the type, and showed the similarity of Nature's methods in them all.

From this sprung Lister's great discovery, and all those processes of modern life-saving surgery which Lister's name suggests; processes which have resulted in revolution in the art, have stripped it of its terrors, and have vastly widened its powers of saving life and alleviating suffering.

By Pasteur the germ was planted, the way was made clear, and surgeons all the world over have occupied themselves in working out the details, to the untold benefit of humanity.

Pasteur, however, again as the great pathfinder, took up new researches, and by his investigations as to the diffusion of silkworm disease, showed not only that an infectious malady could be due to microbes, but demonstrated the influence of their surroundings on the growth of these pathogenic micro-organisms, and the influence of vitality in resisting their development. But again Pasteur showed the way by making it clear that the virulence of the microbes of disease could be diminished, or as the phrase is, attenuated, by appropriate cultivation. On this idea hangs the treatment of hydrophobia, a great thing, but not all, for this idea has been to medicine what the doctrine of fermentation has to surgery—the germ of new methods, the origin of new departures. Applied by Pasteur himself to the prevention of such diseases as anthrax, the possibility of attenuating a virus paved the way to the production of immunity, and thus became the starting point of some of the most striking features of the medicine of to-day. As a patient worker, a laborious, painstaking investigator, he was a model to his fellows, but it is as the genius, the father of ideas, the man who could always see his way when others failed, that Louis Pasteur will be remembered.

